# 

**Published:** 1873-11-01

**Authors:** 


### Medical Examiner.

A Semi-Monthly Journal of Medical Sciences.

No. 21. CHICAGO, NOVEMBER 1, 1873. Vol. XIV.

TERMS.—Yearly Subscriptions, Three Dollars, in advance. Single Numbers Fifteen Cents.
All Communications should be addressed to Publishers Medical Examiner, 65 Randolph Street, corner State, Chicago.

